# The Impact of Acute Psychosocial Stress on Magnetoencephalographic Correlates of Emotional Attention and Exogenous Visual Attention

**DOI:** 10.1371/journal.pone.0035767

**Published:** 2012-06-11

**Authors:** Ludger Elling, Harald Schupp, Janine Bayer, Ann-Kathrin Bröckelmann, Christian Steinberg, Christian Dobel, Markus Junghofer

**Affiliations:** 1 Institute for Biomagnetism and Biosignalanalysis, University Hospital Muenster, Muenster, Germany; 2 Department of Psychology, University of Konstanz, Konstanz, Germany; 3 Department of Systems Neuroscience, University Hospital Hamburg-Eppendorf, Hamburg, Germany; University of Regensburg, Germany

## Abstract

Stress-induced acute activation of the cerebral catecholaminergic systems has often been found in rodents. However, little is known regarding the consequences of this activation on higher cognitive functions in humans. Theoretical inferences would suggest increased distractibility in the sense of increased exogenous attention and emotional attention. The present study investigated the influence of acute stress responses on magnetoencephalographic (MEG) correlates of visual attention. Healthy male subjects were presented emotional and neutral pictures in three subsequent MEG recording sessions after being exposed to a TSST-like social stressor, intended to trigger a HPA-response. The subjects anticipation of another follow-up stressor was designed to sustain the short-lived central catecholaminergic stress reactions throughout the ongoing MEG recordings. The heart rate indicates a stable level of anticipatory stress during this time span, subsequent cortisol concentrations and self-report measures of stress were increased. With regard to the MEG correlates of attentional functions, we found that the N1m amplitude remained constantly elevated during stressor anticipation. The magnetic early posterior negativity (EPNm) was present but, surprisingly, was not at all modulated during stressor anticipation. This suggests that a general increase of the influence of exogenous attention but no specific effect regarding emotional attention in this time interval. Regarding the time course of the effects, an influence of the HPA on these MEG correlates of attention seems less likely. An influence of cerebral catecholaminergic systems is plausible, but not definite.

## Introduction

It has been suggested that, under acute stress, the allocation of attention becomes more automatic and less controlled [1], [2]. A rationale for this suggestion may be derived from a neurobiological perspective. Acute stress elicits a variety of stress responses that can affect attention.

Beyond the stress-induced secretion of glucocorticoids (HPA-response), central chatecholaminergic stress responses (CCR) have been discussed in recent times. One aspect of the CCR is the activation of ascending noradrenergic projections emanating from the locus coeruleus (LC-NE) and the lateral tegmental field [3]. A stress-induced increase in the tonic activity of LC-NE has been demonstrated in a number of animal studies using a variety of stressors [4]. In fact, the LC is one of the most stress-sensitive structures in the brain [5] and, together with the paraventricular nucleus, plays a pivotal role in governing the stress responses.

The LC-NE system is also involved in attention. Specifically, it inhibits spontaneous orienting responses to distracting stimuli and prevents them from disrupting volitionally focused attention [6]. Cortical areas that play a role in spontaneous orienting responses to distractors [7], [8] receive inhibiting phasic input from the LC-NE (see [9], for a review). An increased tonic LC-NE activity may impair this inhibiting phasic input [6]. It is thus plausible that, under acute stress, distractibility is increased and directed attention is impaired [4], [9].

This proposal, however, is largely based on rodent models [10]. In humans, anecdotal evidence from pharmacological practice prevails and there is a lack of controlled trials [3], [11]. The present work intended to substantiate the influence of CCR on two distinct functions of visual attention: namely, emotional attention and exogenous attention. MEG correlates of both respective functions were observed under a state of acute anticipatory stress and compared with a euthymic state.

As a general working hypothesis, we propose that, under anticipatory stress, the direction of attention may be shifted away away from a volitionally controlled direction towards a more spontaneously triggered direction. In terms of experimental operationalization, task-irrelevant but significant stimuli may detract a share of the perceptual resources from task-relevant stimulation in the sense of a biased competion [12], [13]. We expect this biased competition to be reflected by electrophysiological correlates of exogenous attention [14] and also to be reflected by correlates of emotional attention [15], respectively.

### Exogenous Attention

The term “exogenous attention” is defined as attention that is captured by the intrusive salience of an external stimulus [16]. The stimulus salience is usually based on its sudden onset, change or movement within the visual scene [17], [18], or by some other kind of deviance in an otherwise homogeneous stimulus environment. As a common example, flickering banner ads on the Internet exploit this effect.

The macroanatomical circuitry of exogenous attention involves cortico-cortical associations; in particular, associations that emanate from the right temporoparietal junction and the right ventral frontal cortex and innervate extrastriate cortices [7], [19].

Exogenous attention and orienting to a salient visual stimulus is accompanied by an enhanced N1 amplitude [20], [21], reflecting enhanced neural activity in the extrastriate cortex [22].

### Emotional Attention

The term “emotional attention” refers to the spontaneous capture of perceptual processing capacities in response to emotionally significant events in the environment. Such events usually imply the sudden emergence of cues for a pending threat or reward. The allocation of attention is automatic rather than voluntarily controlled. The neuronal network subserving emotional attention involves projections from the central nucleus of the amygdaloid complex (CE) to secondary visual cortices [23]–[25], [19].

An extensively validated electroencephalographic correlate of emotional attention is the Early Posterior Negativity (EPN). Specifically, emotionally significant visual stimuli can elicit a stronger negativity of the evoked potential than can emotionally neutral stimuli [26]–[30]. Typical latencies for the effect vary widely among studies ([31], for review, see [32]). An analogous component (EPNm) occurs in the MEG modality. Extrastriate cortices around the occipital pole have been identified as its main neural generators using fMRI data and distributed MEG source reconstructions [33]–[35]. These extrastriate cortices receive the cholinergic terminals of the emotional attention network.

### Hypotheses

The EPN/EPNm provides a means for quantifying emotional attention and its influence. We presented subjects with task-irrelevant emotional and neutral depictions in the visual periphery along with a competing visual task at the fixation point (see [36] for a related procedure). We expected that emotional depictions would elicit an EPNm in form of an enhanced amplitude of the estimated source signal around the occipital pole when compared to neutral depictions. This constituted a basic prerequisite for the actual investigation. We further hypothesized that this EPNm would be more pronounced under anticipatory stress compared with a euthymic, non-stressful control condition. The visual N1 indicates the orientation towards distracting stimuli in the context of exogenous attention. We hypothesized that anticipatory stress would also increase the N1m response to task-irrelevant visual stimuli in general [37].

## Materials and Methods

### Ethics Statement

The subjects gave their written, informed consent to the testing procedures in advance and received a standard compensation. Prior to each session, the participants were reminded to the option of self-determined termination. It was emphasized that their financial compensation would not be affected then and that criticism or questions would be avoided. The study was part of a transregional collaborative research grant application chaired by the author HTS. Thus, ethics approvals for all projected studies were obtained at the University of Konstanz. In accordance with the guidelines of the Deutsche Forschungsgemeinschaft, an equivalent ethics proposal at the University of Münster was waived.

### Subjects and Screenings

Twenty subjects (with a mean age of 23.1 years; SD  = 3.6) were recruited via postings at the University of Muenster. One subject, was excluded due to abnormal evoked magnetic visual field components. To avoid the possible influence of the ovarian cycle on adrenocortical reactivity, only men were included in the study (see [38], [39] for review). Exclusion criteria (as assessed in an initial telephone screening) included mental disorders according to an abbreviated version of the Structured Clinical Interview for DSM-IV, Axis I [40], left-hand dominance, current medication and habitual smoking. All of the subjects had normal or corrected-to-normal vision.

### General Procedures and Stressors

To avoid any influence of the circadian profiles of adrenocortical reactivity and cognitive ability, all measurements were scheduled to start between 3 and 4 p.m. The subjects were instructed to refrain from consuming alcohol, caffeine, or candy/ample meals 24, 12 and 3 hours, respectively, prior to the measurements and check-ups for compliance were announced. The subjects underwent two procedures on two different days, one with stressor exposition and one as a non-stressful control procedure, in counterbalanced order. Different days of recording are referred to as sessions below. We developed an in-house relayed social role play stress protocol (RESOS), which was derived from stress induction procedures demonstrated by [41], [42]. The protocol included two stressors, of which one was applied prior to the MEG recording and another one was present continuously. The first stressor was based on a classic TSST, but adjusted to the specific requirements of neuroimaging labs (subject immobilization, isolated recording rooms, et cetera). This first stressor was intended to initiate the inert and high-threshold HPA response early enough to develop over time (c.f. [43], [41]). The second stressor was constituted by the tensed anticipation of a similar final TSST-like task. It was intended to sustain a continued anticipatory stress reaction throughout the MEG recording which, in turn, was the main outcome variable. This continued anticipatory reaction was mandatory in order to keep the CCR going on, which otherwise would decay quickly after the offset of a stressor (see also Discussion).

In a stress session, the subject was seated alone in the MEG scanner and underwent an initial resting period of ten minutes while watching a relaxing movie. The subsequent stressor was administered by a board of investigators who addressed the subject via a video screen from outside the room. For the sake of reproducibility and economy, the board was videotaped and played back to all subjects throughout the study (remote). This fact, however, was unknown to the subjects until the final debriefing, and responses to requests were supplied by an additional real staff member.

**Figure 1:**
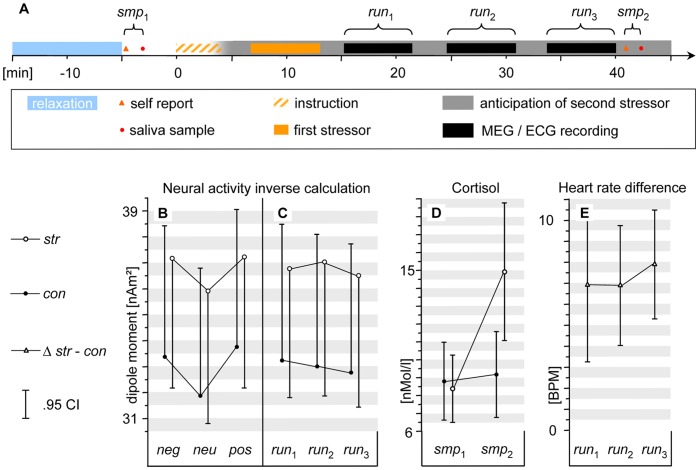
Experimental design and principal findings. Panel A: Timeline of experimental procedures. The onset of the first stressor is defined as the advent of the examination jury giving instructions (dashed orange bar). The solid orange bar refers to the actual self disclosing speech and video feedback. The unsettling situation was not terminated until after the last recording run and sampling were completed in that the subjects anticipated a second stressor. This was announced at the end of the instruction (gray bar). Panels B to E: The closed circles are data points under the stress induction *str*, and the open circles are data points under the control procedure *con*, the open prisms are the differences between both procedures. The vertical bars indicate the.95 confidence limits. Panel B: Bar triplets show activity evoked by aversive (*neg*), neutral (*neu*) and positive images (*pos*) averaged over the subsequent recording runs. Evoked activity refers to the average amplitude of the estimated source strength (mean dipole moments [nAm^2^] derived from the standard ROI and time frame; see Results and Figure 2). Panel C: The same depiction for the three consecutive runs, irrespective of the picture content. Note the stable temporal persistence of the stress main effect. Panel D: Salivary cortisol concentration, sampled prior to (*smp*_1_) and after the stress induction and MEG recordings (*smp*_2_). Panel E: Ipsative data of individual heart rate in corresponding runs (*str* minus *con*). Note, again, the stable stress level over the three subsequent recording runs.

First, the board members introduced themselves as a team of experts in the psychological interpretation of body language, facial expression, emotional prosody and vegetative reactions such as blushing. The presence of polygraph sensors and psychological observation was emphasized to create a feeling of disclosure. Several cameras in the MEG chamber observed the subject at close proximity. The board members, whom the subject was encountering for the first time, acted with formal courtesy and distinct interpersonal distance. The subject was then prompted to convincingly introduce himself as a job candidate in a role-playing manner. The precise nature of the job, however, was not detailed further. Instead, the subject was instructed to omit details regarding professional qualifications and to instead sketch his social skills and his personality. An initial, unrehearsed attempt was to be delivered prior to the MEG measurements. For this initial speech, the subject was given three minutes on a visible timer, during which the remote board remained silent, watching attentively and occasionally taking notes. The board maintained this posture when the subject ended with time remaining. The subjects were instructed not to underrun the time frame. The subject’s performance remained uncommented upon. Instead, the subject saw his own speech as a videotaped feedback to better prepare for a second attempt which the subject was informed would be required after the following MEG recordings. In order to announce an overcharging task demand, the time frame for the second speech was set to eight minutes instead of three minutes as in the initial speech. Subsequently, three consecutive 6∶15-minute runs of MEG and ECG were recorded, separated by two breaks averaging 2∶41 minutes. During the breaks, the subject was reminded to the eventual second stressor. Below, these runs are consecutively referred to as *run*_1_, *run*_2_ and *run*_3_. See figure 1a, for a timeline.

**Figure 2:**
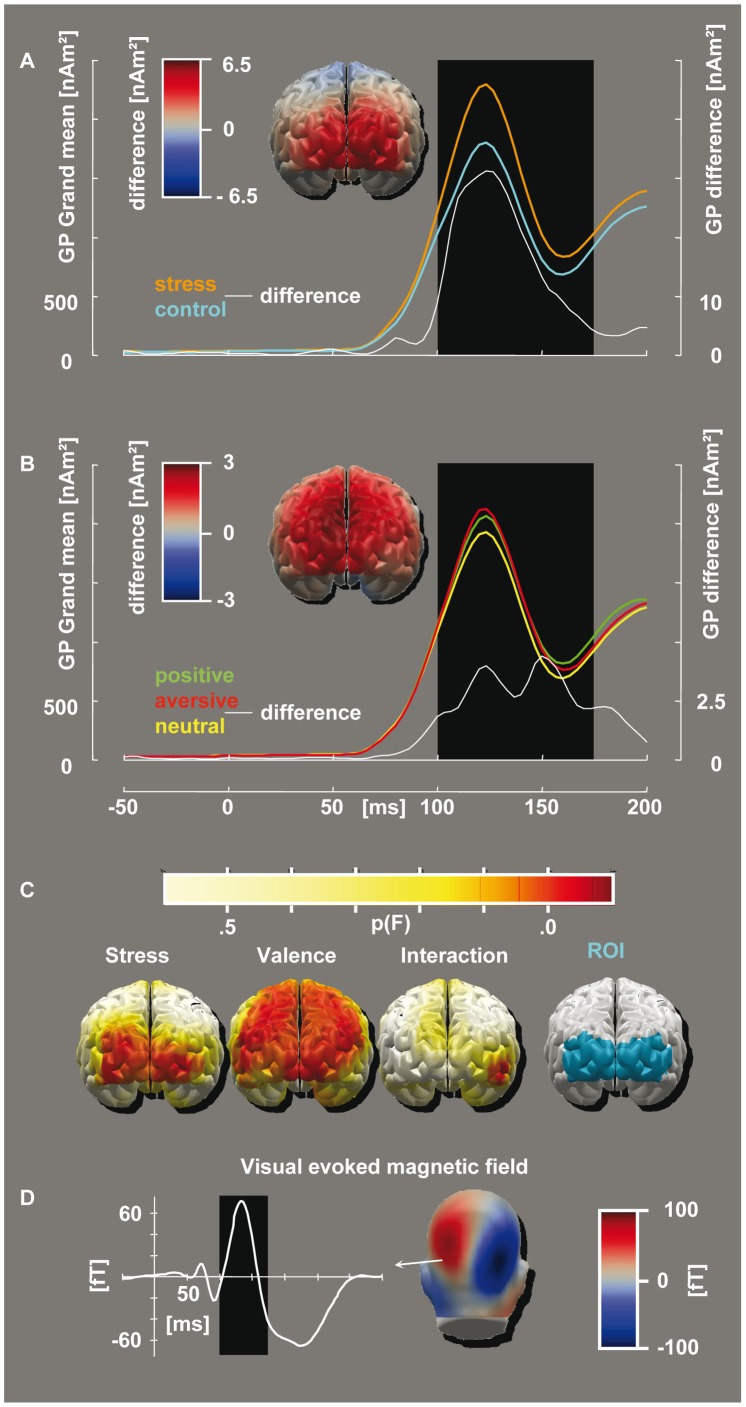
Conditional differences in evoked activity. The morphologies refer to a dipole cluster of interest as depicted in the rightmost image in panel C, whereas the topographies (all from an occipital perspective) pertain to an interval of [100∶175] ms (indicated by the black boxes in Panels A, B and D). The evaluations reported in the Results section are based on this selection of time span and ROI. Panel A: The global power of the estimated dipole moments for the stress induction versus the control procedure (scaling as in the left-hand ordinate) and the corresponding difference (right-hand ordinate, white graph). The topographic depiction also refers to this difference. Panel B: The activity evoked by emotional arousing (positive or aversive) and neutral pictures, as well as the difference between arousing and neutral scenes (topographic depiction and white graph). Panel C: Parametric map of the dipole-wise ANOVA (uncorrected). Left to right: The main effect of stress induction, the main effect of emotional valence category viewed and the interaction between both factors. Shown at the far right is the ROI that was utilized for the area measures. Panel D: The evoked field strength of a selected left occipital SQUID. This depiction allows a comparison between the deflections in Panels A and B with standard visual-evoked fields. The interval of interest, marked as a black box, corresponds to the visual N1m.

The subjects agreed to participate in the evaluation of a psychological assessment for job applicants but were naive to the fact, that the stress itself, and not the utility of the stress interview, was the focus of the experiment. All subjects who were invited after a positive screening to complete the procedures did so without opting for a premature termination. After a standard final debriefing, all subjects judged the procedures to be appropriate in an individual semi-structured interview.

The control session was designed to resemble the stress session with regard to cognitive load and time course, while avoiding stress. Free speech and role-playing was also required, but did not concern a job application. Instead, in the speech, an anonymous acquaintance or a relative of the subject’s choice had to be described in terms of their character traits. Only a single experimenter, who was familiar to the subject from a previous encounter, was present during that session and the cameras were removed. The instructions were delivered by entering the magnetically shielded MEG roomfor a direct dialog. Despite a more cordial interpersonal style, the importance of diligence in this task was emphasized.

### MEG Runs and Visual Stimulation

During the three runs, the subjects were required to watch a continuously present Landolt broken ring that was positioned in the center of the screen with a visual angle of 1.1°. A turn of the ring as a rare target required the reply of pressing a button. The target was not stimulus-locked and could occur at any time. Such a task only permitted a parafoveal processing of the background (see also [44]). Visual tasks overlaying more complex visual scenes (e.g., [34], [15]) have been shown to compete for visual processing resources as early as in the EPN time range (see also [45], [46], [8], [47]–[49]).

During each individual MEG recording, 300 pictures from the International Affective Picture System [50] were presented as a competitive visual context centered on the ring for 600 ms. The pseudo-randomized interstimulus interval varied equally distributed in a [400∶800] ms range. The visual angles of the pictures were 18° in the vertical and 24° in the horizontal directions, respectively. Based on the normative ratings of hedonic valence and emotional arousal [51], the pictures were selected to constitute categories of 100 negative (e.g., violence or mutilations), 100 neutral (e.g., people at daily routine) and 100 positive scenes (e.g., erotica or social affinity). These conditions are referred to, respectively, as *neg*, *neu* and *pos*. The selection provided that the neutral category was equidistant to both emotional categories with respect to both the arousal dimension and valence dimension.

Furthermore, pictures of different emotional categories were matched with respect to a variety of physical stimulus properties (see [52] for the rationale). Specifically, we avoided differences in brightness, contrast, color distribution and complexity.

### Stress-related Measures

A bipolar lead electrocardiogram was recorded simultaneously with the MEG, thus also providing three subsequent datapoints per session (*run*_1_, *run*_2_, *run*_3_). The raw data were evaluated for interbeat interval (R-R) using ANSLAB 2.4 (University of Basel, Institute for Psychology, Switzerland) and averaged per run.

Self-reports were surveyed on computerized visual analog scales that were based on the subscales *RU* and *GS* of the questionnaire *Mehrdimensionaler Befindlichkeitsfragebogen Form A* (MDBF, [53], see also [54], [55]). This instrument was chosen as a German equivalent of the Stress Adjective Checklist (SACL, [56]). The MDBF parallels the SACL with respect to the derived adjective pools and its oft-replicated factor structure of two bipolar subscales in affective introspection [57]. Scale labeling on behalf of the authors translates into English as [agitation:tranquility] and [good mood:bad mood]. The MDBF does not address the term “stress” in a manner that is obvious to the subject. The questionnaire was completed twice per session, after the resting phase and the third MEG run.

Along with the completed MDBF data, the subjects delivered saliva samples (Salivette(R), Sarstedt, Nümbrecht, Germany) that were stored below −18°C and then analyzed for cortisol (CORT) in a single lot. The saliva samples were analyzed by the Institute of Biopsychology at the Technical University Dresden, Germany using a commercially available enzyme-linked immunosorbent assay (IBL International GmbH, Hamburg, Germany) that had a lower detection limit of 0.41 nmol/L. Concealed aliquots confirmed the accuracy of their analyses which had an intra-assay coefficient of variation (c.v.) of 5.06%. The two samplings of both the MDBF and saliva samples are referred to *smp*_1_ and *smp*_2_.

### MEG Recording and Data Preprocessing

During picture presentation, visual evoked magnetic fields were continuously recorded using a whole-head device with 275 first-order axial SQUID gradiometers (Omega 275, CTF, VSM MedTech, Coquitlam, Canada), filtered online (150 Hz low-pass for aliasing, and 50 Hz notch for the European power grid) and sampled at 600 Hz. The continuous data were then band-pass filtered offline in a [0.1∶48] Hz range using a zero-phase second-order Butterworth filter.

The data were then aligned to the stimulus onset and baseline-adjusted using a [−200∶0] ms pre-stimulus interval. The method for the statistical control of artifacts in high density EEG/MEG data was used for single-trial data editing and artifact rejection [58]. This procedure (1) detects individual channel artifacts, (2) detects global artifacts, (3) replaces artifact-contaminated sensors with spline interpolation statistically weighted on the basis of all remaining sensors and (4) computes the variance of the signal across trials to document the stability of the averaged waveform. The rejection of artifact-contaminated trials and sensor epochs relies on the calculation of statistical parameters for the absolute measured magnetic field amplitudes over time, their standard deviation over time, the maximum of their gradient over time (the first temporal derivative) and the determination of the boundaries of each of these three parameters.

After averaging, the cortical sources of the event-related magnetic fields were estimated using the L2-Minimum-Norm-Estimates method (L2-MNE, [59], see also [60]). This inverse modeling technique was applied to reconstruct the topography of the primary current underlying the magnetic field distribution. It allows the estimation of distributed neural network activity without a priori assumptions regarding the location and/or number of current sources [61]. In addition, from all of the possible generator sources, only those that were exclusively determined by the measured magnetic fields were considered. A spherical shell consisting of 350 evenly distributed dipole pairs (pointing in the azimuthal and polar directions) was used as the source model. A source shell radius of 87% of the individually fitted head radius was chosen, which roughly corresponds to the gray matter depth. Although the distributed source reconstruction in MEG does not give the precise location of cerebral generators, it allows for a fairly good approximation of cortical generators and their corresponding assignment to larger cortical structures. Across all of the participants and conditions, a Tikhonov regularization parameter k of 0.2 was applied. The topographies of source direction-independent neural activities (the vector length of the estimated source activities at each position) were calculated for each individual participant, condition and time point based on the averaged magnetic field distributions and the individual sensor positions for each participant and run. To promote better intelligibility, below L2-MNE topographic maps were projected onto a realistic brain geometry.

A time interval of [100∶175] ms and a selection of 20 occipital estimated sources was determined based on both the magnetic flux topographies of the EPNm component as reported in [33] (c.f. Figures 2b, 2c) and a further data-driven restriction as reported in the former place. Given the extraordinarily short latency of the EPNm, the same spatial and temporal selection happened also to be adjusted for the visual N1m also ([62], [31], Figure 2d). The same selected region and time interval of interest were thus used for the observation of both emotional and exogenous attention. The figures and morphologies that are depicted refer to this occipital source cluster (Figure 2c, rightmost) if no exception is stated. All of the MEG evaluation and statistics procedures were performed using the free EMEGS 2.3 software [63] running under MATLAB 7 SP3 (The MathWorks, Natick, MA, USA). Unless stated otherwise, ANOVA results reported below refer to uncorrected degrees of freedom. However, reanalysis using Greenhouse-Geisser correction for lack of sphericity did in no case alter the exceedance of significance thresholds.

## Results

The factor steps are abbreviated as follows: anticipatory stress *str* versus control procedure *con*, saliva samplings plus questionnaire completion, as well as MEG recording runs (consecutively *smp*_1_, *run*_1_, *run*_2_, *run*_3_ and *smp*_2_; see Figure 1a) and picture category (with *neg*, *neu* and *pos* referring to aversive, neutral and appetitive scenes, respectively).

*Manipulation check: Stress-related measures.* On average, during *str*, the heart rate was 7.2 beats per minute faster than during *con* (SD  = 6.1 BPM). This represented an increase by 10.7% that remained markedly stable over the subsequent runs (see Figure 1e). The corresponding main effect of stressor on HR resulted in F_(1,18)_ = 19.6; p<.001.

The cortisol concentrations were similar at all datapoints except for a distinct rise in the sample taken after stress induction (Figure 1d). An a priori guided contrast of all datapoints against this data point was significant ({*smp*_1_ *con*, *smp*_1_ *str*, *smp*_2_ *con*, *smp*_2_ *str*} = {−1, −1, −1, 3}, resulting in F_(1,18)_ = 14.084; p<.01). Expressed by absolute values, the mean cortisol concentration had at a baseline level of 8.4 nmol/L prior to the stress induction and was increased by additional 6.5 nmol/L thereafter.

Regarding the convergent validity of the MDBF subscales, the intercorrelation was r^2^ = 0.874, p<.001, thus confirming the descriptions by the authors [53], and their condition-dependent trends were very similar. Subsequent analyses were thus based on a sum score ranging within [0∶100] (relaxed : stressed). After stress induction at *smp*_2_, self-reported stress was, on average, MN = 14.4 points higher in *str* than in *con* (SD  = 13.2), as opposed to a negligible difference before the induction (MN  = 2.31, SD  = 11.5 at *smp*_1_). The same guided contrast that was previously applied to the saliva samples was significant here as well (F_(1,18)_ = 34.345, p<.001).

The effects of the stressors on the objective stress parameters were not affected by the individual orders of *str* and *con* on subsequent days. With regard to the HR, an interaction of stressor anticipation and the individual order of the stressor conditions yielded non-significant results (F_(1,17)_ = 0.908; p = .354). For the difference of cortisol concentrations *str*-*con*, there was no interaction between samples (pre vs. post) and individual orders (F_(1,17)_ = .075; p = .787). Subjective stress related mood ratings were more enhanced for participants who were exposed to the stressors in the first session compared to subjects being exposed in the second session. For the pooled stress scales, the ipsative differences of *smp*_1_ minus *smp*_2_ in *con* and *str* did significantly interact with the order of stress conditions, (F_(1,17)_ = 7,57; p = .014).

### MEG Data

Emotionally significant pictures elicited a larger visual evoked activity than did neutral pictures (Figure 1b, 2b with {*neg*, *neu*, *pos*} = {1; −2; 1} being highly significant, F_(1,18)_ = 25.003; p<.001. We will refer to this effect as EPNm.

There was not even a minor interaction of the stressor condition and the EPNm (see Figure 2b). Accordingly, when subtractions of the corresponding valence categories between stress and non-stress conditions (*exp* minus *con*) were subjected to a contrast test {*neg*, *neu*, *pos*} = {1; −2; 1}, this was not significant (F_(1,18)_ = 0.986; p = .33).

The visual N1m amplitude increased under anticipatory stress. This was confirmed by a significant main effect of the stress condition with F_(1,18)_ = 7.769; p = .012 (Figure 1c).

The above observations were based on three subsequent runs; however, single runs did not vary noteworthy both regarding the main effect of emotional picture content as well as regarding the main effect of stressor condition. The main effects of emotion and the main effect of stressor condition were stable, even at the level of individual subjects. Specifically, *neu* was subtracted from the mean of the arousing conditions *neg* and *pos*, which was then used to quantify emotional attention. In a cross-correlation of *run*_1_ times *run*_3_, (the runs with the largest temporal distance), the individual consistency was r = .477; p<.05. The temporal course of the main effect of stress was also intraindividually constant. Specifically, the difference *str* minus *con* was subjected to a crosscorrelation *run*_1_ times *run*_3_, resulting in r = 0.863, p<.000.

Both the main effect of stressor condition and the main effect of emotional picture category were independent of treatment order (Stress: F_(1,17)_ = .692; p = .417; Emotion: F_(2,34)_ = .759; p = .476).

The individual N1m difference component (stress minus control) did neither correlate with the cortisol rise after stress induction (post minus pre; r = −.185; p = .45) nor with the individual heart rate difference (stress minus control; r = −.067; p = .78).

## Discussion

### Stressor Verification

As the RESOS is no established stressor protocol, controlling its effectiveness was one prerequisite for our actual investigation. The average HR accelerated by 10.7 percent of the control condition and remained at this elevated level throughout the three MEG runs (over some 24 minutes, see Figures 1a, 1e). This temporal sustainability of the anticipatory stress reaction throughout the MEG recordings was a central prerequisite for our study. Transient and temporally volatile stress reactions, such as the CCR that constitute our theoretical foundation would otherwise not have been able to affect the MEG correlates of attention. One of the central purposes of the RESOS protocol is to resolve this methodological constraint. In a frequent experimental account, stress exposition and experimental testing are purposefully performed subsequently or even time-lagged [64]–[70], but see [71]. This procedure is tailored for investigating effects of stress-related glucocorticoids on cognitive functions, which have an inert rise time and half life. However, investigating the consequences of CCR requires a state of stress at the very moment of MEG/EEG recording [10].

The preceding first stress exposition was intended to also activate slower systems with a higher threshold, i.e. the HPA, during the recording of the MEG. Regarding the HPA, the cortisol reaction lies in between typical values found in the classical Trier Social Stress Test (TSST) and the Montreal Imaging Stress Task (MIST) when examining male subjects in the same age range at late afternoon ([72], [43], see also [73], [74], for a review, see [75], but note the below study limitations regarding the precision of cortisol measurement).

### MEG Effects

Fulfilling another prerequisite, an EPNm-like modulation of visual evoked activity by the affective content of the stimuli was observed (Figures 1b, 2b).

In line with our expectation, we observed a N1m source strength increase under stressor anticipation in our male sample, indicative of an increased influence of exogenous attention. Surprisingly, emotional attention seemed unaffected, given that the EPNm was not affected by the stress state. By contrast, primary evidence for a modulation of emotional attention using a variety of methods was found by [76]–[79] (In [80], this modulation was restricted to clinical populations only). Rather than stress in general, cortisol seems to be a key mediator. Specifically, [78] found this modulation to develop gradually over time after stress exposition, which supports this assumption. Dose-dependant effects of hydrocortison administration on emotional attention were found by [79].

### The Role of CCR

We proposed that CCR would cause the above MEG effects. However, several other factors as part of a full-blown stress response could account for them. In a previous study, we took care to avoid the influence of such confounds, in particular of HPA [10]. Despite this supporting the causal attribution, it goes at the expense of ecological validity. Thus, in the present study, a TSST-like stress protocol prior to the MEG recordings was used to also activate HPA. The more inert HPA should then have gradually increased during the time course of the three MEG recording runs [81]–[84], [79]. However, in the MEG data, we observed utmost stable effects rather than temporal changes (Figure 1c). As the HR increase showed the same temporal stability, we would infer a causal role of more transient stress reactions instead. This is also consistent with our initial posit of CCR. However, this CCR is not restricted to the above LC-NE activation (see [4], for a detailed discussion). Furthermore, fast-acting systemic aspects of the stress response could account for our finding, provided they diffuse through the blood-brain-barrier. For many glandotropic secretagogues, this does not hold [85]–[87], including systemic CRH [88], [89]. and ACTH (87). Nevertheless, further research is needed for a more definite attribution of the present findings.

### Limitations Und Conclusion

Several methodological limitations of this study should be acknowledged. Importantly, for the reasons summarized in [90], we included only male subjects. Note that there are clear sex differences regarding emotional stimulus processing and stress responsivity [91]–[93].

The sample size is adapted to MEG imaging studies. Although this does not compromise the validity of positive findings, this size is rather small for the investigation of the HPA. Potential influences of the HPA on the attentional functions of interest may escape detection. Also with regard to the HPA, the quantification of individual HPA responsivity is based on two saliva samples only. This falls short of the precision obtained by an area measure using a complete time series. What is more, we infer the HPA rise time from the literature [43], [41], [94].

The small sample size may also account for the observation that none of the measures of stress responsivity correlates with the electrophysiological effects discussed above. Regarding these data, no conclusions can be drawn regarding the relative influence of different aspects of the stress response.

It remains unclear why the order of stressor condition and control condition affected the subjective stress reports while no corresponding order effects were found in the HPA, the HR or in the MEG data. Importantly, it remains to be explained why no effects of anticipatory stress on markers of emotional attention were observed.

By contrast, our observations regarding exogenous attention are consistent with a recent EEG study in humans [37]. Corroborating evidence has also been provided by [95], with pharmacological challenges in a behavioral study.

In summary, three conclusions appear reasonable: First, anticipatory stress causes enhanced distractibility in the visual modality (see also our corresponding auditory observations by [10]). Second, this effect seems specific to exogenous attention and no effect on emotional attention was observed. Further research is required to account for this dissociation. Third, this effect does not seem to be based on HPA signalling. A specific role of central catecholaminergic stress reactions is plausible, but needs confirmation.
